# Bis[(cyanido-κ*C*)bis(1,10-phenanthroline-κ^2^ *N*,*N*′)copper(II)] pentakis­(cyanido-κ*C*)nitro­soferrate(II) dimethyl­formamide monosolvate

**DOI:** 10.1107/S1600536812036264

**Published:** 2012-08-25

**Authors:** Olesia V. Kozachuk, Vladimir N. Kokozay, Olga Yu. Vassilyeva, Brian W. Skelton

**Affiliations:** a Department of Inorganic Chemistry, Taras Shevchenko National University of Kyiv, Volodymyrska str. 64, Kyiv 01601, Ukraine; b Department of Inorganic Chemistry II, Ruhr University Bochum, Universitätsstrasse 150, 44780, Bochum, Germany; c Centre for Microscopy, Characterisation and Analysis, University of Western Australia, 35 Stirling Highway, Crawley, WA 6009, Australia

## Abstract

The title compound, [Cu(CN)(C_12_H_8_N_2_)_2_]_2_[Fe(CN)_5_(NO)]·C_3_H_7_NO, is formed of discrete [Cu(phen)_2_CN]^+^ cations (phen is 1,10-phenanthroline), nitro­prusside [Fe(CN)_5_(NO)]^2−^ anions and dimethyl­formamide (DMF) mol­ecules of crystallization. The metal atom has a distorted trigonal–bipyra­midal coordination environment, defined by four N atoms of two phen mol­ecules and a C atom of the cyanide group (in the equatorial position). The [Fe(CN)_5_(NO)]^2−^ anion was found to be disordered about (but not on) a crystallographic twofold rotation axis. Geometries were restrained to ideal values. The dimethyl­formamide solvent mol­ecule was found to be disordered about a crystallographic inversion centre.

## Related literature

For direct synthesis using sodium nitro­prusside, see: Vreshch *et al.* (2009*a* ▶,*b* ▶). For structures containing [Cu(phen)_2_CN]]^+^ cations, see: Dunaj-Jurčo *et al.* (1993 ▶); Potočňák *et al.* (1996*a* ▶,*b* ▶). For a review of structures containing nitro­prusside anions, see: Soria *et al.* (2002 ▶).
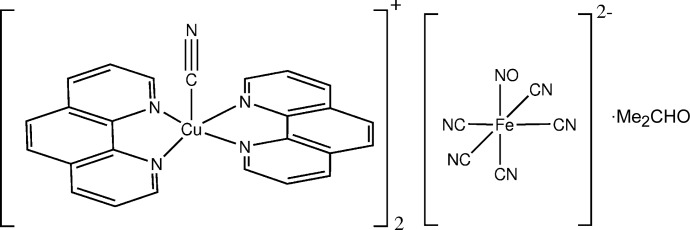


## Experimental

### 

#### Crystal data

[Cu(CN)(C_12_H_8_N_2_)_2_]_2_[Fe(CN)_5_(NO)]·C_3_H_7_NO
*M* *_r_* = 1188.99
Monoclinic, 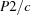

*a* = 11.9235 (16) Å
*b* = 10.7836 (16) Å
*c* = 19.805 (3) Å
β = 97.252 (3)°
*V* = 2526.1 (6) Å^3^
*Z* = 2
Mo *K*α radiation
μ = 1.18 mm^−1^
*T* = 150 K
0.26 × 0.21 × 0.07 mm

#### Data collection

Siemens SMART CCD diffractometer
Absorption correction: multi-scan (*SADABS*; Sheldrick, 1996 ▶) *T*_min_ = 0.86, *T*_max_ = 1
26129 measured reflections
5844 independent reflections
3739 reflections with *I* > 2σ(*I*)
*R*_int_ = 0.066

#### Refinement

*R*[*F* ^2^ > 2σ(*F* ^2^)] = 0.052
*wR*(*F* ^2^) = 0.135
*S* = 1.04
5844 reflections
430 parameters
44 restraints
H-atom parameters constrained
Δρ_max_ = 0.56 e Å^−3^
Δρ_min_ = −0.59 e Å^−3^

### 

Data collection: *SMART* (Siemens, 1995 ▶); cell refinement: *SAINT* (Siemens, 1995 ▶); data reduction: *SAINT*; program(s) used to solve structure: *SIR92* (Altomare *et al.*, 1994 ▶); program(s) used to refine structure: *SHELXL97* (Sheldrick, 2008 ▶); molecular graphics: *ORTEPII* (Johnson, 1976 ▶) and *DIAMOND* (Brandenburg, 1999 ▶); software used to prepare material for publication: *WinGX* (Farrugia, 1999 ▶).

## Supplementary Material

Crystal structure: contains datablock(s) I, global. DOI: 10.1107/S1600536812036264/hg5236sup1.cif

Structure factors: contains datablock(s) I. DOI: 10.1107/S1600536812036264/hg5236Isup2.hkl

Additional supplementary materials:  crystallographic information; 3D view; checkCIF report

## Figures and Tables

**Table 1:** Selected bond lengths (Å)

Cu1—C25	1.964 (4)
Cu1—N3	2.011 (3)
Cu1—N1	2.024 (3)
Cu1—N4	2.090 (3)
Cu1—N2	2.164 (3)
Fe1—N9	1.630 (6)
Fe1—C27	1.933 (7)
Fe1—C26′	1.934 (8)
Fe1—C27′	1.937 (7)
Fe1—C26	1.938 (7)
Fe1—C28	1.959 (6)

**Fig. 1.**
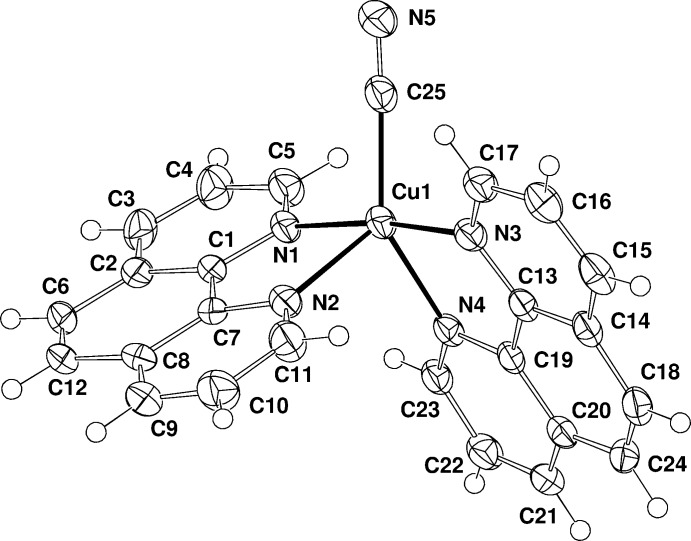
Molecular structure of the cation with the numbering scheme (the non-hydrogen atoms ellipsoids are shown at the 50% probability level).

**Fig. 2.**
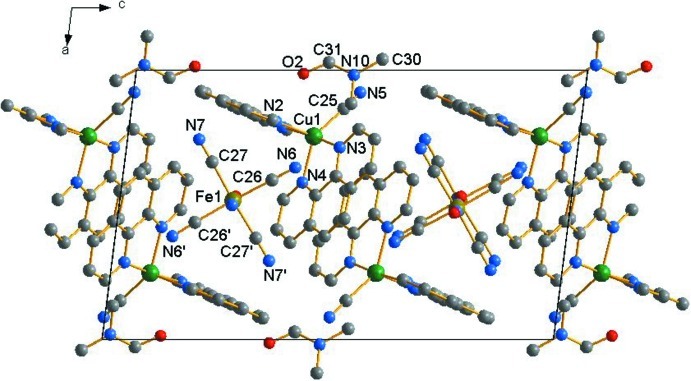
Unit-cell contents of (I). Only one set of each of the disordered atoms is shown for clarity.

## supplementary crystallographic information

### Comment

Recently, we have shown that sodium nitroprusside could be used as a source of metalloligand or could supply the second metal upon its destruction in direct synthesis of heterometallic Cu/Fe complexes (Vreshch *et al.*, 2009*a*,*b*). Within this study we prepared a new cation-anion Cu/Fe complex based on the self-assembly of nitroprusside anion and Cu cation containing a bidentate amine.The title compound was isolated from the solution obtained by reacting copper powder and sodium nitroprusside with (NH_4_)_2_C_2_O_4_ and 1,10-phenanthroline in DMF. Obviously, the nitroprusside partially decomposed to supply a cyanide group to the cation while sodium oxalate did not participate. To the best of our knowledge the title compound has not been structurally characterized.

The title compound, [Cu(C_12_H_8_N_2_)_2_CN]_2_[Fe(CN)_5_(NO)].DMF, is formed of discrete [Cu(phen)_2_CN]^+^ cations (phen is 1,10-phenanthroline), nitroprusside [Fe(CN)_5_(NO)]^2-^ anions and DMF molecules of crystallization. The [Cu(phen)_2_CN]^+^ cation has no crystallographically imposed symmetry (Fig. 1). The metal atom has a distorted trigonal-bipyramidal geometry, coordinating four nitrogen atoms of two phen molecules and a carbon atom of the cyanide group. One nitrogen from each phen ligand coordinates to the copper centre in an equatorial position, with Cu–N distances of 2.090 (3) and 2.164 (3) Å, while the other nitrogen atoms occur at axial positions, with Cu–N distances of 2.011 (3) and 2.024 (3) Å. The cyanide group occupies the remaining equatorial position with the Cu–C bond of 1.964 (4) Å. A similar coordination geometry was observed in other complexes containing [Cu(phen)_2_CN]]^+^ cations (Dunaj-Jurčo *et al.*, 1993; Potočňák *et al.*, 1996*a*,*b*).

The [Fe(CN)_5_(NO)]^2-^ anion was found to be disordered about (but not on) the crystallographic 2-fold axis. Geometries were restrained to ideal values (Soria *et al.*, 2002): a usual distorted octahedral pagoda-like conformation of the nitroprusside anion, with Fe–C bond distances in the range 1.933 (7)–1.959 (6) Å and a Fe–N bond distance of 1.630 (6) Å. There was no indication from the refinement of any disorder between the nitrosyl and cyanide groups.

The structure is completed by dimethylformamide solvent molecules that were found to be disordered about the crystallographic inversion centre.

### Experimental

Copper powder (0.08 g, 1.25 mmol), (NH_4_)_2_C_2_O_4_.H_2_O (0.18 g, 1.25 mmol), Na_2_[Fe(CN)_5_(NO)].2H_2_O (0.37 g, 1.25 mmol), phen.H_2_O (0.75 g, 3.77 mmol), and 20 ml DMF were heated to 323–333 K and magnetically stirred until total dissolution of the copper was observed (50 min). The resulting blue solution was filtered and allowed to stand at room temperature. Blue-green plate-like microcrystals of the title compound were formed after one day. They were collected by filter-suction, washed with dry Pr^i^OH and finally dried *in vacuo* (yield: 20%).

### Refinement

The non-hydrogen atoms were refined anisotropically. Hydrogen atoms were placed at idealized positions (C–H = 0.95 Å, *U*_iso_(H) = 1.2*U*_eq_ C for CH and 1.5*U*_eq_ C for CH_3_) and refined as part of riding models.

### Crystal data

**Table:**

[Cu(CN)(C_12_H_8_N_2_)_2_]_2_[Fe(CN)_5_(NO)]·C_3_H_7_NO	*F*(000) = 1212
*M**_r_* = 1188.99	*D*_x_ = 1.563 Mg m^−^^3^
Monoclinic, *P*2/*c*	Mo *K*α radiation, λ = 0.71073 Å
Hall symbol: -p 2yc	Cell parameters from 4070 reflections
*a* = 11.9235 (16) Å	θ = 2.5–27.6°
*b* = 10.7836 (16) Å	µ = 1.18 mm^−^^1^
*c* = 19.805 (3) Å	*T* = 150 K
β = 97.252 (3)°	Plate, green
*V* = 2526.1 (6) Å^3^	0.26 × 0.21 × 0.07 mm
*Z* = 2	

### Data collection

**Table:**

Siemens SMART CCD diffractometer	3739 reflections with *I* > 2σ(*I*)
Graphite monochromator	*R*_int_ = 0.066
ω scans	θ_max_ = 27.6°, θ_min_ = 1.7°
Absorption correction: multi-scan (*SADABS*; Sheldrick, 1996)	*h* = −15→15
*T*_min_ = 0.86, *T*_max_ = 1	*k* = −14→14
26129 measured reflections	*l* = −25→25
5844 independent reflections	

### Refinement

**Table:**

Refinement on *F*^2^	Primary atom site location: structure-invariant direct methods
Least-squares matrix: full	Secondary atom site location: difference Fourier map
*R*[*F*^2^ > 2σ(*F*^2^)] = 0.052	Hydrogen site location: inferred from neighbouring sites
*wR*(*F*^2^) = 0.135	H-atom parameters constrained
*S* = 1.04	*w* = 1/[σ^2^(*F*_o_^2^) + (0.0315*P*)^2^ + 6.0554*P*] where *P* = (*F*_o_^2^ + 2*F*_c_^2^)/3
5844 reflections	(Δ/σ)_max_ = 0.034
430 parameters	Δρ_max_ = 0.56 e Å^−^^3^
44 restraints	Δρ_min_ = −0.59 e Å^−^^3^

### Special details

**Table:**

Geometry. All s.u.'s (except the s.u. in the dihedral angle between two l.s. planes) are estimated using the full covariance matrix. The cell s.u.'s are taken into account individually in the estimation of s.u.'s in distances, angles and torsion angles; correlations between s.u.'s in cell parameters are only used when they are defined by crystal symmetry. An approximate (isotropic) treatment of cell s.u.'s is used for estimating s.u.'s involving l.s. planes.
Refinement. Refinement of *F*^2^ against ALL reflections. The weighted *R*-factor *wR* and goodness of fit *S* are based on *F*^2^, conventional *R*-factors *R* are based on *F*, with *F* set to zero for negative *F*^2^. The threshold expression of *F*^2^ > 2σ(*F*^2^) is used only for calculating *R*-factors(gt) *etc*. and is not relevant to the choice of reflections for refinement. *R*-factors based on *F*^2^ are statistically about twice as large as those based on *F*, and *R*- factors based on ALL data will be even larger.The Fe(CN)_5_(NO) anion was found to be disordered about (but not on) the crystallographic 2-fold axis. Geometries were restrained to ideal values. There was no indication from the refinement of any disorder between the nitrosyl and cyanide groups. The dimethylformamide solvent molecule was found to be disordered about the crystallographic inversion centre.

### Fractional atomic coordinates and isotropic or equivalent isotropic displacement parameters (Å^2^)

**Table:**

	*x*	*y*	*z*	*U*_iso_*/*U*_eq_	Occ. (<1)
Cu1	0.24634 (4)	0.51817 (4)	0.41187 (2)	0.02860 (12)	
Fe1	0.48386 (19)	1.06768 (8)	0.25085 (16)	0.0258 (5)	0.5
O1	0.4694 (8)	1.3257 (5)	0.2553 (5)	0.064 (4)	0.5
O2	0.0132 (6)	0.0540 (6)	0.3713 (3)	0.0567 (18)	0.5
N1	0.2124 (3)	0.6513 (3)	0.34018 (13)	0.0286 (7)	
N10	0.0187 (9)	0.0109 (9)	0.4835 (4)	0.046 (3)	0.5
N2	0.1909 (3)	0.4049 (3)	0.32377 (14)	0.0314 (7)	
N3	0.2866 (3)	0.3710 (3)	0.47243 (14)	0.0281 (7)	
N4	0.4172 (2)	0.5023 (3)	0.39952 (13)	0.0258 (6)	
N5	0.0827 (3)	0.6436 (3)	0.50242 (15)	0.0407 (8)	
N6	0.3618 (10)	1.0421 (12)	0.3797 (5)	0.034 (3)	0.5
N7	0.2577 (7)	1.0334 (7)	0.1589 (4)	0.0325 (15)	0.5
N6'	0.6170 (11)	1.0517 (13)	0.1269 (6)	0.038 (3)	0.5
N7'	0.7084 (7)	1.0498 (7)	0.3477 (4)	0.0325 (15)	0.5
N8	0.5	0.7807 (4)	0.25	0.0437 (13)	
N9	0.4757 (8)	1.2186 (5)	0.2510 (7)	0.039 (3)	0.5
C1	0.1684 (3)	0.6090 (3)	0.27799 (16)	0.0276 (8)	
C2	0.1320 (3)	0.6898 (4)	0.22391 (17)	0.0321 (9)	
C3	0.1418 (3)	0.8171 (4)	0.23649 (19)	0.0399 (10)	
H3	0.1176	0.8747	0.2014	0.048*	
C4	0.1865 (4)	0.8588 (4)	0.29959 (19)	0.0406 (10)	
H4	0.1939	0.9451	0.3086	0.049*	
C5	0.2207 (3)	0.7723 (4)	0.35009 (18)	0.0358 (9)	
H5	0.2515	0.8016	0.3938	0.043*	
C6	0.0857 (3)	0.6375 (4)	0.16002 (17)	0.0355 (9)	
H6	0.0587	0.6914	0.1236	0.043*	
C7	0.1600 (3)	0.4779 (4)	0.26856 (16)	0.0279 (8)	
C8	0.1175 (3)	0.4299 (4)	0.20479 (17)	0.0334 (9)	
C9	0.1135 (3)	0.3004 (4)	0.19781 (19)	0.0414 (10)	
H9	0.0873	0.2637	0.1551	0.05*	
C10	0.1477 (4)	0.2278 (4)	0.2532 (2)	0.0462 (11)	
H10	0.1458	0.14	0.2493	0.055*	
C11	0.1856 (4)	0.2834 (4)	0.3154 (2)	0.0404 (10)	
H11	0.2085	0.2317	0.3535	0.048*	
C12	0.0793 (3)	0.5150 (4)	0.15008 (17)	0.0366 (9)	
H12	0.0494	0.4834	0.1067	0.044*	
C13	0.3944 (3)	0.3303 (3)	0.47224 (15)	0.0251 (8)	
C14	0.4385 (3)	0.2252 (3)	0.50800 (16)	0.0283 (8)	
C15	0.3657 (4)	0.1628 (3)	0.54723 (17)	0.0353 (9)	
H15	0.3916	0.0922	0.5734	0.042*	
C16	0.2570 (3)	0.2047 (4)	0.54740 (18)	0.0360 (9)	
H16	0.2071	0.1625	0.5733	0.043*	
C17	0.2198 (3)	0.3097 (3)	0.50953 (17)	0.0323 (8)	
H17	0.1445	0.3378	0.5104	0.039*	
C18	0.5514 (3)	0.1872 (3)	0.50234 (17)	0.0329 (9)	
H18	0.5813	0.1155	0.5261	0.039*	
C19	0.4645 (3)	0.3996 (3)	0.43208 (15)	0.0248 (7)	
C20	0.5760 (3)	0.3616 (3)	0.42840 (16)	0.0280 (8)	
C21	0.6399 (3)	0.4355 (4)	0.38890 (17)	0.0329 (9)	
H21	0.7161	0.414	0.3848	0.039*	
C22	0.5925 (3)	0.5385 (4)	0.35627 (17)	0.0344 (9)	
H22	0.6354	0.5886	0.3295	0.041*	
C23	0.4811 (3)	0.5690 (3)	0.36269 (16)	0.0290 (8)	
H23	0.4491	0.6405	0.3398	0.035*	
C24	0.6164 (3)	0.2517 (3)	0.46361 (17)	0.0327 (9)	
H24	0.6907	0.2233	0.4597	0.039*	
C25	0.1439 (3)	0.5939 (4)	0.47038 (17)	0.0321 (9)	
C26	0.4078 (9)	1.0528 (11)	0.3314 (4)	0.024 (2)	0.5
C27	0.3411 (6)	1.0476 (9)	0.1938 (4)	0.021 (2)	0.5
C28	0.5	0.8869 (5)	0.25	0.0358 (13)	
C26'	0.5654 (10)	1.0548 (15)	0.1727 (5)	0.036 (3)	0.5
C27'	0.6237 (6)	1.0557 (10)	0.3120 (4)	0.025 (2)	0.5
C29	0.1247 (8)	−0.0542 (8)	0.4854 (5)	0.052 (3)	0.5
H29A	0.1101	−0.141	0.4725	0.078*	0.5
H29B	0.1655	−0.0504	0.5315	0.078*	0.5
H29C	0.1704	−0.0155	0.4534	0.078*	0.5
C30	−0.0397 (12)	0.0140 (12)	0.5423 (6)	0.057 (4)	0.5
H30A	−0.0972	0.0794	0.5369	0.086*	0.5
H30B	0.0143	0.031	0.5828	0.086*	0.5
H30C	−0.0762	−0.0663	0.5475	0.086*	0.5
C31	−0.0278 (8)	0.0603 (8)	0.4248 (4)	0.041 (2)	0.5
H31	−0.0975	0.1032	0.4245	0.049*	0.5

### Atomic displacement parameters (Å^2^)

**Table:**

	*U*^11^	*U*^22^	*U*^33^	*U*^12^	*U*^13^	*U*^23^
Cu1	0.0344 (2)	0.0324 (2)	0.01888 (19)	0.0065 (2)	0.00289 (16)	0.00409 (18)
Fe1	0.0389 (13)	0.0173 (4)	0.0198 (4)	−0.0003 (5)	−0.0017 (8)	0.0003 (7)
O1	0.126 (11)	0.023 (3)	0.042 (4)	0.005 (4)	0.004 (6)	−0.001 (3)
O2	0.058 (4)	0.066 (4)	0.046 (3)	0.011 (3)	0.002 (3)	0.012 (3)
N1	0.0343 (16)	0.0327 (17)	0.0184 (13)	0.0077 (13)	0.0020 (12)	0.0013 (12)
N10	0.058 (6)	0.026 (4)	0.051 (8)	0.004 (4)	−0.005 (5)	−0.007 (5)
N2	0.0377 (18)	0.0337 (18)	0.0225 (14)	0.0085 (14)	0.0029 (13)	−0.0008 (12)
N3	0.0329 (16)	0.0299 (16)	0.0219 (13)	0.0018 (13)	0.0045 (12)	0.0005 (12)
N4	0.0327 (15)	0.0280 (16)	0.0170 (12)	0.0004 (13)	0.0038 (11)	0.0025 (11)
N5	0.047 (2)	0.048 (2)	0.0287 (16)	0.0075 (17)	0.0081 (15)	0.0032 (15)
N6	0.042 (5)	0.041 (6)	0.018 (4)	0.003 (4)	−0.003 (3)	−0.003 (3)
N7	0.046 (4)	0.034 (2)	0.0199 (19)	0.000 (2)	0.013 (2)	−0.002 (2)
N6'	0.051 (6)	0.030 (5)	0.034 (5)	0.003 (4)	0.009 (4)	0.008 (4)
N7'	0.046 (4)	0.034 (2)	0.0199 (19)	0.000 (2)	0.013 (2)	−0.002 (2)
N8	0.077 (4)	0.026 (3)	0.030 (2)	0	0.013 (2)	0
N9	0.059 (9)	0.030 (3)	0.024 (2)	−0.002 (3)	−0.011 (6)	0.003 (4)
C1	0.0256 (18)	0.040 (2)	0.0178 (15)	0.0086 (16)	0.0039 (13)	0.0015 (14)
C2	0.0289 (19)	0.047 (2)	0.0215 (16)	0.0100 (17)	0.0068 (14)	0.0053 (15)
C3	0.040 (2)	0.047 (2)	0.0320 (19)	0.0116 (19)	0.0015 (17)	0.0159 (17)
C4	0.050 (3)	0.033 (2)	0.037 (2)	0.0067 (19)	−0.0013 (19)	0.0092 (17)
C5	0.046 (2)	0.036 (2)	0.0258 (18)	0.0053 (18)	0.0026 (16)	0.0016 (15)
C6	0.030 (2)	0.059 (3)	0.0176 (16)	0.0094 (18)	0.0041 (14)	0.0062 (16)
C7	0.0246 (17)	0.040 (2)	0.0202 (15)	0.0106 (16)	0.0047 (13)	0.0003 (15)
C8	0.036 (2)	0.044 (2)	0.0221 (16)	0.0108 (17)	0.0086 (15)	−0.0037 (15)
C9	0.041 (2)	0.053 (3)	0.0297 (19)	0.008 (2)	0.0016 (17)	−0.0131 (18)
C10	0.059 (3)	0.039 (2)	0.041 (2)	0.010 (2)	0.003 (2)	−0.0064 (19)
C11	0.054 (3)	0.032 (2)	0.033 (2)	0.0095 (19)	−0.0014 (18)	−0.0014 (16)
C12	0.0321 (19)	0.061 (3)	0.0177 (15)	0.0105 (19)	0.0051 (14)	−0.0018 (17)
C13	0.0331 (19)	0.0252 (18)	0.0164 (14)	0.0010 (15)	0.0008 (13)	−0.0005 (13)
C14	0.039 (2)	0.0250 (18)	0.0190 (15)	0.0028 (16)	−0.0022 (15)	−0.0023 (13)
C15	0.055 (3)	0.026 (2)	0.0234 (17)	−0.0027 (18)	0.0004 (17)	−0.0010 (14)
C16	0.048 (2)	0.035 (2)	0.0256 (18)	−0.0057 (18)	0.0058 (17)	0.0033 (15)
C17	0.038 (2)	0.035 (2)	0.0246 (17)	0.0030 (17)	0.0047 (15)	0.0024 (15)
C18	0.045 (2)	0.0261 (19)	0.0246 (17)	0.0094 (17)	−0.0070 (16)	−0.0033 (14)
C19	0.0320 (19)	0.0264 (18)	0.0150 (14)	0.0034 (15)	−0.0013 (13)	−0.0016 (13)
C20	0.0333 (19)	0.032 (2)	0.0185 (15)	0.0021 (16)	0.0003 (14)	−0.0050 (14)
C21	0.0302 (19)	0.046 (2)	0.0229 (17)	0.0018 (17)	0.0046 (15)	−0.0065 (15)
C22	0.037 (2)	0.042 (2)	0.0244 (17)	−0.0032 (18)	0.0071 (15)	0.0006 (16)
C23	0.034 (2)	0.033 (2)	0.0197 (16)	0.0007 (16)	0.0023 (14)	0.0022 (14)
C24	0.033 (2)	0.037 (2)	0.0257 (17)	0.0119 (17)	−0.0040 (15)	−0.0038 (15)
C25	0.034 (2)	0.039 (2)	0.0228 (17)	−0.0005 (17)	0.0019 (15)	0.0036 (15)
C26	0.034 (5)	0.015 (4)	0.022 (4)	0.009 (4)	0.000 (4)	−0.003 (3)
C27	0.030 (4)	0.025 (4)	0.011 (3)	0.005 (4)	0.013 (3)	0.002 (3)
C28	0.059 (4)	0.034 (3)	0.014 (2)	0	0.002 (2)	0
C26'	0.040 (6)	0.039 (6)	0.026 (5)	−0.007 (5)	−0.008 (4)	0.007 (4)
C27'	0.033 (5)	0.026 (4)	0.020 (4)	−0.003 (4)	0.018 (4)	0.002 (3)
C29	0.061 (6)	0.032 (5)	0.058 (6)	0.014 (4)	−0.015 (5)	−0.004 (4)
C30	0.072 (8)	0.048 (7)	0.047 (7)	−0.015 (6)	−0.007 (6)	−0.008 (5)
C31	0.048 (5)	0.040 (5)	0.034 (4)	0.010 (4)	−0.002 (4)	0.014 (3)

### Geometric parameters (Å, º)

**Table:**

Cu1—C25	1.964 (4)	C6—C12	1.336 (6)
Cu1—N3	2.011 (3)	C6—H6	0.95
Cu1—N1	2.024 (3)	C7—C8	1.399 (5)
Cu1—N4	2.090 (3)	C8—C9	1.403 (6)
Cu1—N2	2.164 (3)	C8—C12	1.449 (5)
Fe1—N9	1.630 (6)	C9—C10	1.367 (6)
Fe1—C27	1.933 (7)	C9—H9	0.95
Fe1—C26'	1.934 (8)	C10—C11	1.394 (5)
Fe1—C27'	1.937 (7)	C10—H10	0.95
Fe1—C26	1.938 (7)	C11—H11	0.95
Fe1—C28	1.959 (6)	C12—H12	0.95
O1—N9	1.161 (7)	C13—C14	1.403 (5)
O2—C31	1.224 (10)	C13—C19	1.434 (5)
N1—C5	1.322 (5)	C14—C15	1.407 (5)
N1—C1	1.355 (4)	C14—C18	1.425 (5)
N10—C31	1.334 (12)	C15—C16	1.373 (6)
N10—C30	1.430 (15)	C15—H15	0.95
N10—C29	1.442 (13)	C16—C17	1.399 (5)
N2—C11	1.321 (5)	C16—H16	0.95
N2—C7	1.360 (4)	C17—H17	0.95
N3—C17	1.326 (5)	C18—C24	1.350 (5)
N3—C13	1.358 (4)	C18—H18	0.95
N4—C23	1.330 (4)	C19—C20	1.402 (5)
N4—C19	1.367 (4)	C20—C21	1.407 (5)
N5—C25	1.158 (5)	C20—C24	1.427 (5)
N6—C26	1.167 (11)	C21—C22	1.371 (5)
N7—C27	1.147 (9)	C21—H21	0.95
N6'—C26'	1.159 (12)	C22—C23	1.390 (5)
N7'—C27'	1.160 (10)	C22—H22	0.95
N8—C28	1.145 (7)	C23—H23	0.95
C1—C2	1.406 (5)	C24—H24	0.95
C1—C7	1.428 (5)	C29—H29A	0.98
C2—C3	1.397 (6)	C29—H29B	0.98
C2—C6	1.431 (5)	C29—H29C	0.98
C3—C4	1.371 (6)	C30—H30A	0.98
C3—H3	0.95	C30—H30B	0.98
C4—C5	1.390 (5)	C30—H30C	0.98
C4—H4	0.95	C31—H31	0.95
C5—H5	0.95		
			
C25—Cu1—N3	95.49 (13)	C8—C7—C1	119.7 (3)
C25—Cu1—N1	92.01 (13)	C7—C8—C9	117.5 (3)
N3—Cu1—N1	171.89 (11)	C7—C8—C12	119.0 (4)
C25—Cu1—N4	142.30 (13)	C9—C8—C12	123.6 (3)
N3—Cu1—N4	80.91 (11)	C10—C9—C8	119.2 (4)
N1—Cu1—N4	95.03 (11)	C10—C9—H9	120.4
C25—Cu1—N2	124.13 (14)	C8—C9—H9	120.4
N3—Cu1—N2	93.50 (11)	C9—C10—C11	119.6 (4)
N1—Cu1—N2	79.68 (12)	C9—C10—H10	120.2
N4—Cu1—N2	93.58 (11)	C11—C10—H10	120.2
N9—Fe1—C27	93.7 (5)	N2—C11—C10	122.9 (4)
N9—Fe1—C26'	96.3 (7)	N2—C11—H11	118.6
C27—Fe1—C26'	91.1 (5)	C10—C11—H11	118.6
N9—Fe1—C27'	96.4 (5)	C6—C12—C8	120.6 (3)
C27—Fe1—C27'	169.4 (3)	C6—C12—H12	119.7
C26'—Fe1—C27'	90.9 (5)	C8—C12—H12	119.7
N9—Fe1—C26	92.7 (6)	N3—C13—C14	123.7 (3)
C27—Fe1—C26	90.1 (4)	N3—C13—C19	116.8 (3)
C26'—Fe1—C26	170.9 (3)	C14—C13—C19	119.5 (3)
C27'—Fe1—C26	86.3 (4)	C13—C14—C15	116.5 (3)
N9—Fe1—C28	177.7 (4)	C13—C14—C18	119.4 (3)
C27—Fe1—C28	87.9 (3)	C15—C14—C18	124.1 (3)
C26'—Fe1—C28	82.1 (5)	C16—C15—C14	119.5 (3)
C27'—Fe1—C28	82.0 (3)	C16—C15—H15	120.3
C26—Fe1—C28	88.9 (4)	C14—C15—H15	120.3
C5—N1—C1	118.7 (3)	C15—C16—C17	120.1 (4)
C5—N1—Cu1	126.3 (2)	C15—C16—H16	120
C1—N1—Cu1	114.8 (2)	C17—C16—H16	120
C31—N10—C30	120.6 (9)	N3—C17—C16	121.9 (4)
C31—N10—C29	118.9 (8)	N3—C17—H17	119
C30—N10—C29	120.4 (8)	C16—C17—H17	119
C11—N2—C7	118.0 (3)	C24—C18—C14	120.8 (3)
C11—N2—Cu1	131.7 (3)	C24—C18—H18	119.6
C7—N2—Cu1	110.2 (2)	C14—C18—H18	119.6
C17—N3—C13	118.3 (3)	N4—C19—C20	123.3 (3)
C17—N3—Cu1	127.5 (3)	N4—C19—C13	116.7 (3)
C13—N3—Cu1	114.1 (2)	C20—C19—C13	120.0 (3)
C23—N4—C19	117.8 (3)	C19—C20—C21	116.6 (3)
C23—N4—Cu1	130.8 (2)	C19—C20—C24	118.8 (3)
C19—N4—Cu1	111.2 (2)	C21—C20—C24	124.6 (3)
O1—N9—Fe1	175.9 (13)	C22—C21—C20	120.1 (4)
N1—C1—C2	122.1 (3)	C22—C21—H21	120
N1—C1—C7	117.7 (3)	C20—C21—H21	120
C2—C1—C7	120.3 (3)	C21—C22—C23	119.4 (3)
C3—C2—C1	117.5 (3)	C21—C22—H22	120.3
C3—C2—C6	124.0 (3)	C23—C22—H22	120.3
C1—C2—C6	118.5 (4)	N4—C23—C22	122.8 (3)
C4—C3—C2	119.9 (4)	N4—C23—H23	118.6
C4—C3—H3	120	C22—C23—H23	118.6
C2—C3—H3	120	C18—C24—C20	121.5 (3)
C3—C4—C5	118.7 (4)	C18—C24—H24	119.2
C3—C4—H4	120.6	C20—C24—H24	119.2
C5—C4—H4	120.6	N5—C25—Cu1	176.4 (3)
N1—C5—C4	123.1 (3)	N7—C27—Fe1	178.2 (9)
N1—C5—H5	118.5	N8—C28—Fe1	174.28 (7)
C4—C5—H5	118.5	N6'—C26'—Fe1	176.9 (14)
C12—C6—C2	121.9 (3)	N7'—C27'—Fe1	178.7 (9)
C12—C6—H6	119	O2—C31—N10	124.4 (9)
C2—C6—H6	119	O2—C31—H31	117.8
N2—C7—C8	122.8 (4)	N10—C31—H31	117.8
N2—C7—C1	117.4 (3)		

## References

[bb1] Altomare, A., Cascarano, G., Giacovazzo, C., Guagliardi, A., Burla, M. C., Polidori, G. & Camalli, M. (1994). *J. Appl. Cryst.* **27**, 435.

[bb2] Brandenburg, K. (1999). *DIAMOND* Crystal Impact GbR, Bonn, Germany.

[bb3] Dunaj-Jurčo, M., Potočňák, I., Cíbik, J., Kabešová, M., Kettmann, V. & Mikloš, D. (1993). *Acta Cryst.* C**49**, 1479–1482.

[bb4] Farrugia, L. J. (1999). *J. Appl. Cryst.* **32**, 837–838.

[bb5] Johnson, C. K. (1976). *ORTEPII* Report ORNL-5138. Oak Ridge National Laboratory, Tennessee, USA.

[bb6] Potočňák, I., Dunaj-Jurčo, M. & Mikloš, D. (1996*a*). *Acta Cryst.* C**52**, 2406–2409. 10.1107/s0108270100020503 11313559

[bb7] Potočňák, I., Dunaj-Jurčo, M., Mikloš, D. & Jäger, L. (1996*b*). *Acta Cryst.* C**52**, 48–50. 10.1107/s0108270100020503 11313559

[bb8] Sheldrick, G. M. (1996). *SADABS* University of Gottingen, Germany.

[bb9] Sheldrick, G. M. (2008). *Acta Cryst.* A**64**, 112–122. 10.1107/S0108767307043930 18156677

[bb10] Siemens (1995). *SMART* and *SAINT* Siemens Analytical X-ray Instruments Inc., Madison, Wisconsin, USA.

[bb11] Soria, D. B., Villalba, M. E. C., Piro, O. E. & Aymonino, P. J. (2002). *Polyhedron*, **21**, 1767–1774.

[bb12] Vreshch, O. V., Nesterova, O. V., Kokozay, V. N., Dyakonenko, V. V., Shishkin, O. V., Cormary, B., Malfant, I. & Jezierska, J. (2009*a*). *Z. Anorg. Allg. Chem.* **635**, 2316–2323.

[bb13] Vreshch, O. V., Nesterova, O. V., Kokozay, V. N., Skelton, B. W., Garcia, C. J. G. & Jezierska, J. (2009*b*). *Inorg. Chem. Commun.* **12**, 890–894.

